# Engineering of a chromogenic enzyme screening system based on an auxiliary indole‐3‐carboxylic acid monooxygenase

**DOI:** 10.1002/mbo3.795

**Published:** 2019-01-21

**Authors:** Vida Časaitė, Mikas Sadauskas, Justas Vaitekūnas, Renata Gasparavičiūtė, Rita Meškienė, Izabelė Skikaitė, Mantas Sakalauskas, Jevgenija Jakubovska, Daiva Tauraitė, Rolandas Meškys

**Affiliations:** ^1^ Department of Molecular Microbiology and Biotechnology, Institute of Biochemistry, Life Sciences Center Vilnius University Vilnius Lithuania

**Keywords:** aldehyde dehydrogenase, amidohydrolase, functional screening, indole‐3‐carboxylic acid monooxygenase, metagenomics

## Abstract

Here, we present a proof‐of‐principle for a new high‐throughput functional screening of metagenomic libraries for the selection of enzymes with different activities, predetermined by the substrate being used. By this approach, a total of 21 enzyme‐coding genes were selected, including members of xanthine dehydrogenase, aldehyde dehydrogenase (ALDH), and amidohydrolase families. The screening system is based on a pro‐chromogenic substrate, which is transformed by the target enzyme to indole‐3‐carboxylic acid. The later compound is converted to indoxyl by a newly identified indole‐3‐carboxylate monooxygenase (Icm). Due to the spontaneous oxidation of indoxyl to indigo, the target enzyme‐producing colonies turn blue. Two types of pro‐chromogenic substrates have been tested. Indole‐3‐carboxaldehydes and the amides of indole‐3‐carboxylic acid have been applied as substrates for screening of the ALDHs and amidohydrolases, respectively. Both plate assays described here are rapid, convenient, easy to perform, and adaptable for the screening of a large number of samples both in *Escherichia coli* and *Rhodococcus* sp. In addition, the fine‐tuning of the pro‐chromogenic substrate allows screening enzymes with the desired substrate specificity.

## 1 INTRODUCTION

In light of the growing importance of biocatalysis, strategies that provide improvements in screening of novel enzymes are of considerable interest. Among other enzymes, aldehyde dehydrogenases (ALDHs), especially exhibiting a broad substrate spectrum, are potential biocatalysts for biotechnology and are applicable in the detoxification of aldehydes, generated during metabolism of different natural and xenobiotic compounds (Kotchoni, Kuhns, Ditzer, Kirch, & Bartels, 2006; Lyu et al., 2017; Singh et al., 2014).

Metagenomics, which helps to circumvent the cultivation of bacteria and select genes directly from the environment, has become a powerful tool in search of new enzymes and metabolic pathways for the industrial biotechnology over the past decades (Allen, Moe, Rodbumrer, Gaarder, & Handelsman, 2009; Maruthamuthu, Jiménez, Stevens, & Elsas, 2016; Suenaga, Ohnuki, & Miyazaki, 2007; Varaljay et al., 2016). Many studies show that the function‐based screening or selection approaches permits an effective identification of different biocatalysts, such as lipases/esterases (Reyes‐Duarte, Ferrer, & García‐Arellano, 2012), cellulases (Maruthamuthu et al., 2016), and oxygenases (Nagayama et al., 2015), from diverse environmental sources and microbial habitats. However, the common problem in the search for new enzymes is the absence of an appropriate screening system. Usually, the functional screening of desired activities is based on chromogenic approach including the formation of blue indigo pigment, fluorogenic substrates, and/or sensors (Kennedy et al., 2011; Rüther, 1980; Seok et al., 2018; Shang, Chan, Wong, & Liao, 2018; Ye, Peng, Niu, Luo, & Zhang, 2018). Notwithstanding that several chromogenic substrates such as indole, indole carboxylic acids, and indole‐3‐carboxaldehyde applicable for plate and other high‐throughput (HTP) assays have been developed and applied for screening various dioxygenases and broad substrate range monooxygenases (Celik, Speight, & Turner, 2005; Choi et al., 2003; Eaton & Chapman, 1995; Ensley et al., 1983; Furuya, Takahashi, Ishii, Kino, & Kirimura, 2004; McClay, Boss, Keresztes, & Steffan, 2005; O'Connor, Dobson, & Hartmans, 1997; Shi et al., 2013; Willetts, Joint, Gilbert, Trimble, & Mühling, 2012), a limited number of HTP methods for detection of other oxidoreductases, for example, aldehyde dehydrogenases, have been elaborated (Chen et al., 2014; Oyobiki et al., 2014; Reisinger et al., 2006; Seok et al., 2018; Wexler, Bond, Richardson, & Johnston, 2005). Moreover, those approaches are too restricted for a special substrate, cannot be used in a plate format or require sophisticated equipment.

The aim of this study was to develop a novel platform for the functional screening of the enzymes, particularly ALDHs. First, we searched for indole‐3‐carboxylic acid (I3CA)‐degrading microorganisms and corresponding genes in metagenomes to determine whether any could transform I3CA to indigo. We have successfully identified Icm encoding gene, which was used for the creation of the screening method. By using the developed approach, we succeeded in a screening of diverse ALDHs with a broad substrate specificity. Furthermore, the auxiliary Icm enzyme was applied for screening of amidohydrolases using the amide of indole‐3‐carboxylic acid as a substrate. The Icm was active both in Gram‐negative and Gram‐positive bacteria, and hence, the enzyme was suitable for a functional screening of enzymes in different hosts.

## 2 MATERIALS AND METHODS

### 2.1 Chemicals

Chemicals used in this study are listed in Table A1. Gel resins were purchased from GE Healthcare (Little Chalfont, UK). Restriction endonucleases and DNA polymerases were from Thermo Fisher Scientific (Vilnius, Lithuania). All reagents used in this study were of analytical grade.

### 2.2 Bacterial strains, plasmids and media

The bacterial strains and plasmids used in this study are listed in Table 1. *Escherichia coli *and *Rhodococcus erythropolis *SQ1 cells were routinely grown in Luria–Bertani (LB) medium at 16–37°C. The following reagents were added to media as needed: IPTG, 40 μg/ml; ampicillin (Ap), 50 μg/ml; chloramphenicol (Cm), 20 µg/ml; kanamycin (Km) 50 μg/ml; tetracycline (Tc), 20 µg/ml; derivatives of I3CA, 1 mM.

**Table 1:** Strains, plasmids, and primers used in this study

	Relevant characteristics	Source
Strain		
* Escherichia coli* DH5α	endA1, gyrA96, hsdR17, recA1, relA1, supE44, thi‐1,Δ(lacZYA‐argF)U169, Φd80lacZΔM15	Sambrook et al. (1989)
*E. coli* BL21(DE3)	F**^−^**, ompT, hsdSB (rB**^−^**, mB**^−^**), dcm, gal, λ(DE3)	Novagen
*Rhodococcus erythropolis* SQ1	Wild‐type strain	Quan and Dabbs (1993)
*Bosea* sp. KVIA	Soil isolate	This study
Plasmid		
pET‐21c (+)	Expression vector Ap^r^, f1, pBR322 ori	Novagen
pET‐28c (+)	Expression vector Km^r^,f1, pBR322 ori	Novagen
pASK‐IBA3	Ap^r^ expression vector, *Strep*‐tag®, f1 origin	IBA lifesciences
pUC19	Ap^r^, cloning vector pMB1ori	Thermo Fisher
pACYC184	Cm^r^, Tc^r^ cloning vector p15A ori	Thermo Fisher
pNitQC1	Ap^r^ (*E. coli*), Cm^r^ (*R. erythropolis* SQ1)	Nakashima and Tamura (2004a)
pNitRT1	Ap^r^ (*E. coli*), Tc^r^ (*R. erythropolis* SQ1)	
pKVIABam8	pUC19 cloned Icm gene, Ap^r^	This study
pACYC‐KVIA	pACYC184 cloned Icm gene, Cm^r^	This study
pET21‐KVIA	pET21a cloned Icm gene, Ap^r^	This study
pET28‐KVIA	*N*‐terminal His_6_‐tagged Icm, Km^r^	This study
pET28‐MBP‐KVIA	*N*‐terminal MBP‐tagged Icm, Km^r^	This study
pASK‐IBA3‐KVIA	*N*‐terminal Strep‐tagged Icm, Ap^r^	This study
pET21‐MO13	C‐terminal His_6 _tag Amidohydrolase gene, Ap^r^	This study
pNitQC1‐KVIA	*icm *gene for expression in *Rhodococcus* sp.	This study
pNitRT1‐Vmix	Vmix ALDH gene for expression in *Rhodococcus* sp	This study
Primer, 5′−3′		
MBP_R_Nco	TTCCATGGGCCCCTGGAACAG	This study
MBP_F	GTGAGCGGATAACAATTCC	This study
KviaEcoR	GAGAATTCGCCATAGATCAGGACC	This study
KviaNde2F	TACATATGAAGGTCATCATCGTAG	This study
Kvia‐IBA3‐F	GAGCGCGGTCTCGAATGAAGGTCATCATC	This study
Kvia‐IBA3‐R	CTGCGAGGTCTCAGCGCTGGACCGCCGCGC	This study
VmixNdeF	TACATATGAGTGCGAACGATATTAAAAC	This study
VmixHindR	CCAAGCTTCAGAACGGAAACCCGC	This study
am13F	GCCATATGGAAAAATCATCATTAC	This study
am13R2	ACTCGAGCCTGGGATTAATAG	This study

### 2.3 General DNA manipulation

Plasmid preparation, restriction endonuclease digestion, DNA ligation, agarose gel electrophoresis, and other standard recombinant DNA techniques were carried out by standard methods (Sambrook, Fritsch, & Maniatis, 1989). DNA sequencing and primer synthesis were performed commercially at the Macrogen (the Netherlands). DNA sequences were analyzed with a BLAST program available at the National Center for Biotechnology Information web site (http://blast.ncbi.nlm.nih.gov/Blast.cgi). Evolutionary analyzes were conducted in MEGA7 (Kumar, Stecher, & Tamura, 2016).

### 2.4 Screening of soil samples and gene cloning

About 1 g of soil samples were suspended in 1 ml 0.9% w/v NaCl solution, and 50 μl aliquots were spread on the agar plates supplemented with 1 mM I3CA. The plates were incubated at 30°C for 48 hr and were subsequently visually inspected for colonies producing the blue indigo pigment. Chromosomal DNA was isolated from the blue pigment producing bacteria, digested with the PstI restriction endonuclease and ligated in the pUC19 vector. *Escherichia coli* DH5α was used for screening of blue colonies on the plates supplemented with 1 mM I3CA.

For the screening assay, the pKVIABam8 encoding the *icm* gene was digested with BamHI and PscI and subcloned to the BamHI and PagI restriction sites of pACYC184 vector and resulted plasmid was designated pACYC‐KVIA. For construction of expression vectors, *icm *gene was PCR‐amplified with primers KviaEcoR and KviaNde2F (Table 1) and pKviaBam8 as a DNA template. All PCR amplifications were performed using Phusion High‐Fidelity PCR Master Mix. PCR product was digested with NdeI/XhoI restriction endonucleases and ligated into pET‐21a(+) previously digested with the corresponding enzymes to obtain pET21‐KVIA. N‐terminal His_6_‐tag was added by subcloning of the *icm* gene into pET28c(+), resulting in pET28‐KVIA. For expression in *R. erythropolis* SQ1, the digested PCR fragment was ligated into pNitQC1 resulting in plasmid pNit‐KVIA. To obtain N‐terminal fusion of Icm with maltose‐binding protein (MBP), *malE *was amplified with primers MBP_F and MBP_R_Nco, digested with XbaI/NcoI, and ligated into pET28‐KVIA resulting in pET28‐MBP‐KVIA. To obtain N‐terminal fusion with Strep‐Tag, Icm encoding gene was amplified with primers Kvia‐IBA3‐F and Kvia‐IBA3‐R, digested with Eco31I and ligated into Eco31I‐digested pASK‐IBA3, resulting in pASK‐IBA3‐KVIA. For cloning of aldehyde dehydrogenase *Vmix* gene, it was PCR‐amplified with primers VmixHindR and VmixNdeF (Table 1) and DNA from the metagenome clone Vmix as template. PCR product was digested with NdeI/HindIII restriction endonucleases and ligated into pNitRT1 previously digested with the corresponding enzymes to obtain pNitRT‐Vmix. For construction of C‐terminal His_6_‐tagged amidohydrolase, *MO13 *gene was PCR‐amplified with primers am13F and am13R2 (Table 1) and pMO13 as DNA matrix. PCR product was digested with NdeI/XhoI restriction endonucleases and ligated into pET‐21a(+) previously digested with the corresponding enzymes to obtain pET21‐MO13. Electrocompetent cells were prepared as described previously (Nakashima & Tamura, 2004b; Stanislauskiene et al., 2012) and used for transformation.

### 2.5 Construction of the metagenomic library and screening for enzymes

For the construction of environmental DNA libraries, surface soils (0–15 cm) from a different fields in district Vilnius (Lithuania) were collected. The environmental DNA was isolated from samples using ZR Soil Microbe DNA Kit (Zymo Research), partially digested with the endonucleases PstI or HindIII and ligated in the pUC19 vector. To analyze the number of clones in the library, quality of the library (a ratio of white/blue colonies), and the average insert length, *E. coli* DH5α cells were transformed with ligation mixtures and spread on LB agar plates supplemented with ampicillin, 1 mM IPTG, and 1 mM X‐gal. Eight white colonies‐forming clones from each library were chosen for plasmid DNA isolation and analysis of the length of the insert. For functional screening, *E. coli *DH5α cells harboring pACYC‐KVIA were transformed with the metagenomic libraries and plated on LB agar plates containing Ap, Cm, as needed and 1 mM solution of derivative I3CA. The plates were incubated at 37°C for 2 days and were subsequently screened for colonies that were able to produce the blue pigment indigo by visual detection. The positive clones were subjected for DNA sequencing. The sequences obtained in the present study were deposited to the GenBank database under the accession numbers MG770119–MG770138, MG786188, MG786189, MG775032, MK284926, and MH476458. The full list is given in Table A2.

### 2.6 Expression and purification of the recombinant proteins

For gene expression, *E. coli *BL21 (DE3) were transformed with pET21‐KVIA, pET28‐KVIA, pET28‐MBP‐KVIA, pASK‐IBA3‐KVIA, and pET21‐MO13. The cells were grown at 30°C with rotary shaking until OD600 reached 0.8, and gene expression was induced with 0.05–0.5 mM IPTG for pET plasmids and 200 μg/L anhydrotetracycline for pASK‐IBA3 plasmid. The cells were incubated at 16–30°C for either 4 hr or overnight, collected by centrifugation, suspended in lysis buffer (50 mM Tris–HCl, pH 8.0, containing 150 mM of NaCl), disrupted by sonication and the lysates were used as total protein sample, while centrifugation‐clarified lysates (16,000 *g* for 10 min) were treated as a soluble fraction. The recombinant proteins were analyzed with 12% denaturing SDS–PAGE.

Purification through His_6_‐tag was carried out with nickel HisTrap™ HP column according to the manufacturer's instructions. Strep‐tagged protein was purified with Strep‐Tactin XT Starter Kit according to manufacturer's protocol. MBP‐fused protein was purified by MBP‐starch affinity chromatography using commercial grade cationic starch‐packed column essentially as described in (Duong‐Ly & Gabelli, 2015). Protein quantification was performed by densitometry with GelAnalyzer software (Pavel & Vasile, 2012) using different concentrations (100, 250, and 500 μg/ml) of bovine serum albumin (ThermoFisher Scientific) as standard.

### 2.7 Bioconversion of aldehydes or carboxylic acids by whole cells

The *E. coli* or *R. erythropolis* SQ1 cells transformed with the appropriate plasmids were grown aerobically in LB containing appropriated antibiotic at 30°C until optical density reached 0.8 (A_600_), then 0.5 mM of IPTG was added and cells were grown aerobically at 30°C for 12 hr. Cells were harvested by centrifugation, washed with 50 mM potassium phosphate buffer (pH 7.2), suspended in the same buffer and used as the whole cells. Then, 1 mM solutions of substrates were added, and bioconversion reactions were carried out at 30°C with shaking at 180 rpm for 1–24 hr. The conversion was followed by changes in UV absorption spectrum in 200–400 nm range or by HPLC/MS analysis, as described previously (Stankevičiūtė et al., 2016).

### 2.8 Monooxygenase activity assay

The monooxygenase activity was evaluated from the decrease of the absorbance at 340 nm due to oxidation of NADH or NADPH (ε_340_ = 6,220 M/cm), using spectrophotometer and was performed at room temperature. Simultaneously, reaction mixtures were incubated overnight at 30°C and inspected for the formation of blue precipitate. A total reaction volume of 1 ml contained 50 mM Tris–HCl, pH 7.5, 1 mM I3CA, different amounts (1–20 mM) of NADH or NADPH and 50 μM of flavin (FAD, FMN or riboflavin). Reactions were initiated by adding 2.5 μg of the purified enzyme or 20 μl of the soluble fraction (approx. 10 μg of total protein).

### 2.9 Aldehyde dehydrogenase activity assay

For colorimetric assay, the cells were disrupted by sonication and the cell‐free extracts were used to analyze the ALDH activity as described in (Bianchi et al., 2017). In brief, the obtained supernatants were mixed with NAD^+^ (200 μM) and NADP^+^ (200 μM), nitroblue tetrazolium chloride (NBT, 200 μM), phenazine methosulfate (PMS, 20 μM), and an appropriate aldehyde (200 μM) in 50 mM Tris–HCl buffer, pH 8.0, at 30°C. A total reaction volume of 200 µl contained 50 µl of cell lysates (approx. 20 μg of total protein), and the reaction was followed spectrophotometrically (*λ* = 580 nm) in 96‐well microtiter plates by monitoring the production of formazan dye after 1 and 3 hr.

### 2.10 Amidohydrolase activity assay

A total reaction volume of 0.5 ml contained 50 mM Tris–HCl, pH 8.5, and 1 mM of appropriate substrate. Reactions were initiated by adding 2.5 μg of the purified enzyme. The progress of the reaction was followed by changes in UV absorption spectrum in 200–600 nm range or by HPLC/MS analysis, as described previously (Stankevičiūtė et al., 2016).

### 2.11 Synthesis of N‐(3‐hydroxypropyl)‐indole‐3‐carboxamide

A solution of indole‐3‐carboxylate (100 mg, 0.62 mmol) and *N,N,N′,N*′‐tetramethyl‐*O*‐(1*H*‐benzotriazol‐1‐yl)uronium hexafluorophosphate (HBTU, 235.3 mg, 0.62 mmol) in dimethylformamide (1.24 ml) was vigorously stirred for 30 min at room temperature. Then, 3‐amino‐1‐propanol (46.6 mg, 0.62 mmol) and triethylamine (86.5 µl, 0.62 mmol) were added to the reaction mixture and continued stirring for additional 12 hr at the same temperature. The reaction mixture was diluted with water (10 ml) and extracted with ethyl acetate (3 × 15 ml). The organic phase was dried (Na_2_SO_4_) and the solvent evaporated under reduced pressure. The residue was purified by column chromatography (silica gel, chloroform/methanol mixture). Yield 65 mg (48%). Synthesized derivative was characterized by NMR spectroscopy and HPLC/MS analysis. NMR spectra were recorded in DMSO‐*d_6_* on a Bruker Ascend 400: ^1^H NMR–400 MHz, ^13^C NMR–100 MHz. Chemical shifts (δ) are reported in ppm relative to the solvent resonance signal as an internal standard. MS (ESI^+^): *m/z *219 [M+H]^+^, 217 [M−H]^−^.

^1^H NMR (DMSO‐*d_6_*): δ = 1.64–1.74 (m, 2H, CH_2_), 3.32 (dd, 2H, *J* = 12.8, 6.7 Hz, CH_2_), 3.48 (dd, 2H, *J* = 12.7, 6.4 Hz, CH_2_), 4.52 (bs, 1H, OH), 7.06–7.18 (m, 2H, CH), 7.42 (d, 1H, *J* = 7.8 Hz, CH), 7.87 (t, 1H, *J* = 5.5 Hz, NH), 7.99 (d, 1H, *J* = 2.9 Hz, CH), 8.13 (d, 1H, *J* = 7.7 Hz, CH), 11.52 (s, 1H, NH). ^13^C NMR (DMSO‐*d_6_*): δ = 33.31, 46.23, 59.15, 111.22, 112.22, 120.68, 121.42, 122.22, 126.52, 128.00, 136.57, 165.19.

## 3 RESULTS AND DISCUSSION

### 3.1 Cloning and identification of indole‐3‐carboxylate monooxygenase

**Figure 1:**
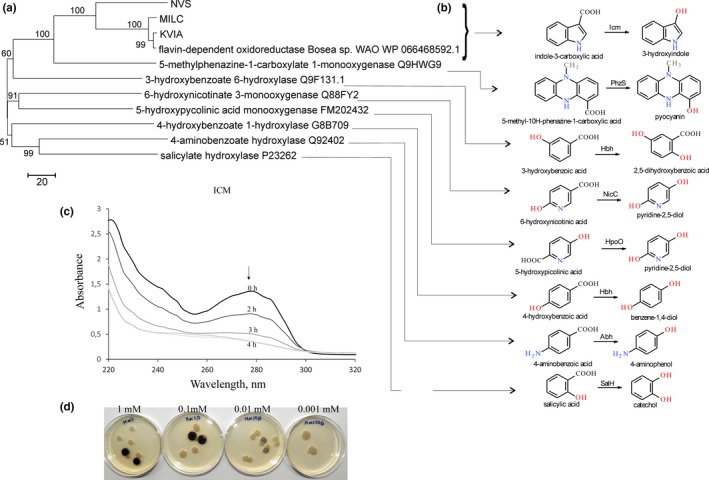
Characterization of Icm‐KVIA. (a) Evolutionary relationship of decarboxylating flavin‐dependent oxidoreductases. The evolutionary history was inferred using the Neighbor‐Joining method (Saitou & Nei, 1987), the evolutionary distances were computed using the number of differences method (Nei & Kumar, 2000) and are in the units of the number of amino acid differences per sequence. The percentage of replicate trees in which the associated taxa clustered together in the bootstrap test are shown next to the branches (Felsenstein, 1985). The tree is drawn to scale, with branch lengths in the same units as those of the evolutionary distances used to infer the phylogenetic tree. (b) Hydroxylation reactions performed by Icm‐related enzymes. SalH, salicylate‐1‐hydroxylase (EC 1.14.13.1); Hbh, 4‐hydroxybenzoate hydroxylase (EC 1.14.13.64); Abh, 4‐aminobenzoate 1‐monooxygenase (EC 1.14.13.27); PhzS, 5‐methylphenazine‐1‐carboxylate 1‐monooxygenase (EC 1.14.13.218); NicC, 6‐hydroxynicotinate 3‐monooxygenase (EC 1.14.13.114); HpoO, 5‐hydroxypicolinate monooxygenase; Icm‐KVIA, I3CA monooxygenase. (c) Time‐course of consumption of I3CA by Icm producing *Escherichia coli* cells. Primary spectrum is black; spectra after two, three, and four hours are depicted in brightening gray. (d) Colonies of *E. coli *DH5α on the plates supplemented with varied concentration of I3CA, blue colonies contain pACYC‐KVIA plasmid, white colonies—an empty pACYC184 vector

To screen enzymes displaying an indigo‐forming activity in the presence of I3CA, two approaches were used. Initially, several blue colonies‐forming bacteria were screened using soil samples and the agar plates supplemented with I3CA. One of these isolates, KVIA, was chosen for further studies. The analysis of the 16S rRNA gene sequence (GenBank accession No. MG775032) revealed that the bacteria belonged to the *Bosea* genus. The genomic library of *Bosea* sp. KVIA was constructed, and the positive clone harboring the plasmid pKVIABam8 was identified based on the ability to form blue colonies on the plates supplemented with I3CA. The nucleotide sequence analysis showed one 1,242 bp long ORF in the insert. The ORF encoded a 414 aa long protein, which was 98% identical to the hypothetical flavin‐dependent oxidoreductase from *Bosea* sp. WAO (GenBank accession No. WP_066468592). Two additional blue colonies‐forming clones were selected from the metagenomic libraries on I3CA agar plates. Both hits, named MILC and NVS, encoded the proteins, which shared 95.7% and 62.7% identity to the protein encoded by the pKVIABam8 plasmid, respectively. According to the sequence analysis, all three screened proteins (KVIA, MILC and NVS clones) belonged to the group A of flavin monooxygenases, which depend on NAD(P)H as external electron donor and contain a glutathione reductase (GR‐2) type Rossmann fold (GXGXXG) for FAD binding. Moreover, several conserved motifs such as DGX_5_R, and GDAX_10_GX_6_DX_3_L characteristic for monooxygenases were identified (Huijbers, Montersino, Westphal, Tischler, & Berkel, 2014). Some dioxygenases such as cumate and *m*‐toluate dioxygenases convert indole‐2‐carboxylic acid and I3CA to indigo. The dioxygenases incorporate two atoms of molecular oxygen, leading to the formation of 2,3‐dihydroxyindoline‐3‐carboxylate. Subsequent reactions are spontaneous and lead to the mixture of indigo, isatin, and indirubin. Moreover, those enzymes are also active toward indole (Eaton & Chapman, 1995). In contrast, the enzymes encoded by the KVIA, MILC, and NVS clones were unrelated to any known dioxygenase and showed the highest sequence similarity to the experimentally characterized monooxygenases such as 5‐methylphenazine‐1‐carboxylate 1‐monooxygenase from *Pseudomonas aeruginosa* PAO1 and 3‐hydroxybenzoate‐6‐hydroxylase from *Pseudomonas alcaligenes* or *Klebsiella oxytoca*. Moreover, the identified enzymes were not active toward indole since the clones did not form colored colonies in the presence of this substrate. In addition, no substrate consumption was observed (HPLC‐MS analysis) when nicotinic, 2‐ and 4‐picolinic, 5‐hydroxypiperazine‐2‐carboxylic, salicylic acid, indoline‐2‐carboxylic, indole‐2‐carboxylic, indole‐4‐carboxylic, indole‐5‐carboxylic, indole‐6‐carboxylic, and indole‐7‐carboxylic were used as substrates for indole‐3‐carboxylate monooxygenase (Icm). We also tested this enzyme with 5‐nitroindole‐3‐carboxylic, 7‐methylindole‐3‐carboxylic, 1‐methylindole‐3‐carboxylic and as well as indole‐3‐carboxaldehyde, indole‐3‐carbonitrile or methyl ester of indole‐3‐carboxylic acid for formation of the blue colonies on plates. No color changes were observed using these derivatives of indole‐3‐carboxylic acid. Based on sequence analysis and substrate specificity, we designated the identified enzyme as an *i*ndole‐3‐*c*arboxylate *m*onooxygenase (Icm). We proposed that Icm performed an oxidative decarboxylation reaction like other known flavin‐dependent monooxygenases that catalyze the decarboxylative hydroxylation of aromatic carboxylic acids (Figure 1b) such as the salicylate monooxygenase from *Pseudomonas putida* (Uemura et al., 2016), 6‐hydroxynicotinic acid 3‐monooxygenases NicC from *P. putida* and *Bordetella bronchiseptica* (Hicks et al., 2016), 5‐methyl phenazine‐1‐carboxylate‐1‐monooxygenase PhzS from *P. aeruginosa* (Mavrodi et al., 2001), 4‐hydroxybenzoate 1‐hydroxylase from *Candida parapsilosis* (Van Berkel, Eppink, Middelhoven, Vervorrt, & Rietjens, 1994), 4‐aminobenzoate monooxygenase from *Agaricus bisporus* (Tsuji, Ogawa, Bando, & Sasaoka, 1986). The relationship between similar enzymes is shown in the phylogenetic tree (Figure 1a).

### 3.2 Expression, protein purification, and characterization of the Icm

To characterize Icm in more detail, the gene encoding Icm was cloned to several expression plasmids, fusing it to His_6_‐Tag, Strep‐Tag, maltose‐binding protein (MBP) or glutathione S‐transferase (GST) or without any tag for protein expression. Also, the plasmid (pNitQC1‐KVIA) for protein expression in *R. erythropolis *SQ1 cells was created. Only the N‐terminal fusion of Icm with MBP (His_6_‐MBP‐His_6_‐Icm) resulted in partially soluble protein (Table A3). Conventional optimization strategies (variation of temperature, inductor concentration, cell density, expression host, buffer composition, etc.) did not result in significant improvement of protein solubility. Once outside the cell, the activity of Icm diminished. No in vitro activity was detected with the purified His_6_‐MBP‐His_6_‐Icm by using different flavin cofactors and following the oxidation of either NADH or NADPH. Similarly, neither substrate consumption nor any intermediate products were detected by HPLC/MS, and no blue precipitate was formed in these in vitro reactions.

**Figure A1:**
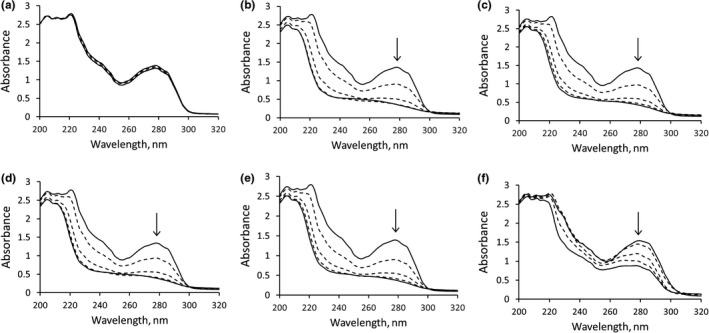
Whole‐cell consumption of I3CA by recombinant cells with Icm. (a) *Escherichia coli *BL21 (DE3) cells (negative control), (b) *E. coli *BL21 (DE3) cells with Icm, (c) cells with His_6_‐Icm, (d) cells with His_6_‐MBP‐His_6_‐Icm, (e) cells with Strep‐Tag MBP‐His_6_‐Icm, (f) *Rhodococcus erythropolis* SQ1 cells with Icm. Consumption was monitored hourly during a period of 5 hr

Since the active purified protein could not be obtained, further work was carried out using the whole cells of recombinant *E. coli *or *R. erythropolis *SQ1 bacteria. It was found that I3CA was consumed by all Icm derivatives at a similar rate (Figure A1). The amount of a blue precipitate formed during the bioconversion of I3C corresponded to the consumption of this substrate. Meanwhile, no pigment appeared in the control reactions, in which the cells transformed with blank vectors were used. This indicates that Icm is active inside the cell and is involved in the conversion of I3CA to indigo blue.

### 3.3 Application of Icm as an auxiliary enzyme for functional screening of aldehyde dehydrogenases

**Figure 2:**
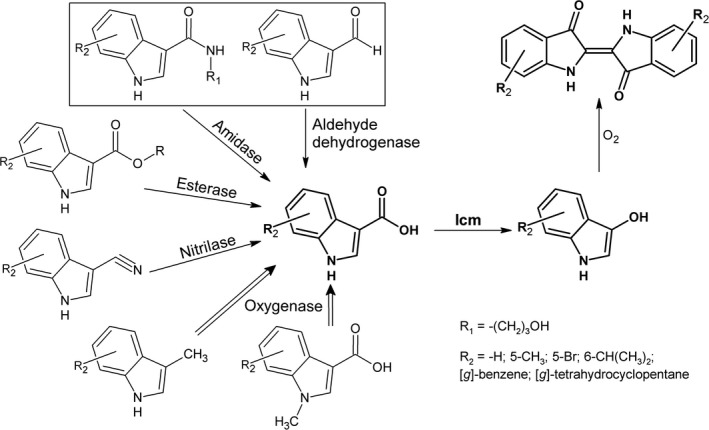
The principal scheme of the functional screening of enzymes based on an auxiliary Icm enzyme. The experimentally tested substrates are boxed. R: any radical

Despite the fact that Icm activity was not detected in vitro, *E. coli* cells harboring the *icm* gene readily produced a blue indigo dye on the agar plates supplemented with I3CA. This property was further exploited to create a system for a functional screening of metagenomic libraries. The idea was to use the appropriate substrate, for example indole‐3‐carboxaldehyde, which would be converted to I3CA by the target enzyme, in this case ALDH. Then, Icm as an auxiliary enzyme would oxidize I3CA into indigo; hence, the colored *E. coli* colonies would indicate the presence of the active ALDH (Figure 2). To test such screening platform, the *icm *gene was subcloned into the pACYC184 vector, compatible with the pUC19, which was used for creation of metagenomic DNA libraries. The *E. coli* DH5α cells transformed with pACYC‐KVIA produced blue colonies on the agar plates supplemented with I3CA (0.01 mM of I3CA in the medium was sufficient for the formation of blue pigment (Figure 1d), but only white colonies were observed when indole‐3‐carboxaldehyde was used as a substrate. Therefore, this strain was further used for screening of metagenomic libraries.

**Figure 3:**
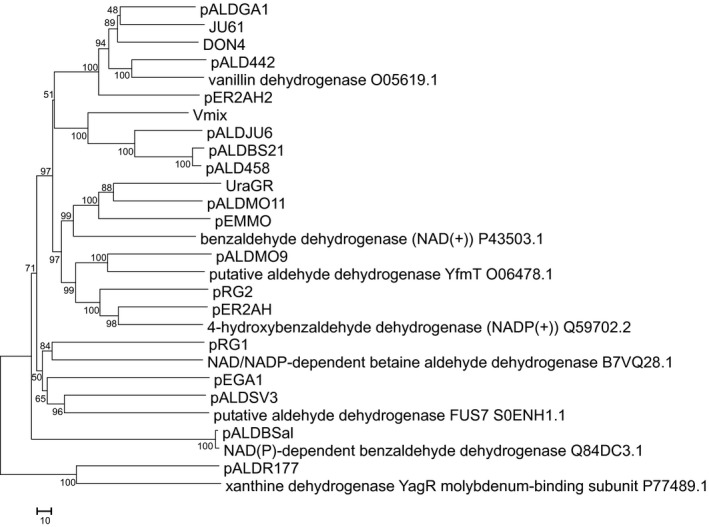
Sequence diversity of metagenomic aldehyde dehydrogenases. The evolutionary history was inferred using the Neighbor‐Joining method (Saitou & Nei, 1987), the evolutionary distances were computed using the number of differences method (Nei & Kumar, 2000) and are in the units of the number of amino acid differences per sequence. The percentage of replicate trees in which the associated taxa clustered together in the bootstrap test are shown next to the branches (Felsenstein, 1985). The tree is drawn to scale, with branch lengths in the same units as those of the evolutionary distances used to infer the phylogenetic tree. The optimal tree with the sum of branch length = 2,362.75195313 is shown. All positions containing gaps and missing data were eliminated. A total of 351 positions in the final dataset were used for data analysis

Twenty‐one metagenomic libraries were created using the pUC19 plasmid and DNA isolated from soil. Each library contained clones with inserts of ~3–15 kb average size, yielding approximately 0.5 Gb of total cloned genomic DNA per library. In order to screen for ALDH activity, about 30,000 clones per library were spread on LB agar supplemented with indole‐3‐carboxaldehyde. In this way, 52 indigo‐forming clones were identified. The clones producing indigo without the presence of Icm (the false positives, i.e., most of such clones encoded Baeyer–Villiger monooxygenases, data not shown) as well as redundant clones were omitted resulting in 20 unique hits harboring the distinct genomic fragments. The sequence analysis of the screened ALDH‐positive clones revealed the presence of genes encoding the proteins that were 73%–99% identical to the known sequences in the NCBI databank and homologous to ALDHs (19 clones), and molybdopterin xanthine dehydrogenase (one clone; see Table A2). Thus, the proposed functional screening approach was suitable for identification of hits expressing ALDHs (Table 2). To gain insight into the phylogenetic relationship of all selected enzymes, the phylogenetic tree was constructed (Figure 3). As revealed by comparison between UniProtKB/SwissProt sequences, nine ALDHs, that is, pDON4, pALDGA1, JU61, pALD442, pER2AH2, Vmix, pALDJU6, pALDBS21, and pALD458 were closest to vanillin dehydrogenase, pALDMO9 was related to *B. subtilis* vanillin dehydrogenase. pEMMO, pALDMO11, and UraGR were related to NAD(+)‐dependent benzaldehyde dehydrogenase and pALDBSal to NAD(P)‐dependent benzaldehyde dehydrogenase. The sequences of clones pRG1, pEGA1, and pALDSV3 were closest to betaine aldehyde dehydrogenase. Also, two 4‐hydroxybenzaldehyde dehydrogenase‐like enzymes were selected (pER2AH, pRG2).

**Table 2:** Functional annotation of clones with aldehyde dehydrogenase activity

Clone	Protein length, aa	The nearest homolog, accession no	Identity, %
DON4	482	Salicylaldehyde dehydrogenase *Betaproteobacteria bacterium* OGA51247.1	77
JU61	507	Salicylaldehyde dehydrogenase *Hydrogenophaga *sp. Root209 WP_056264373	97
pALD442	483	Salicylaldehyde dehydrogenase *Cupriavidus *sp. BIS7 WP_019448853	89
pALD458	484	Phenylacetaldehyde dehydrogenase *Alcaligenes faecalis* WP_060185347	99
pALDBS21	515	Phenylacetaldehyde dehydrogenase *Alcaligenes faecalis* WP_045929579	96
pALDBSal	436	NAD(P)‐dependent benzaldehyde dehydrogenase *Pseudomonas putida* WP_016501743	99
pALDGA1	483	Salicylaldehyde dehydrogenase *Afipia massiliensis* WP_046830129	91
pALDJU6	488	Phenylacetaldehyde dehydrogenase *Pseudomonas *sp. MIACH WP_053136087	96
pALDMO9	485	Aldehyde dehydrogenase *Bacillus thermoamylovorans* WP_041902008	82
pALDMO11	487	Benzaldehyde dehydrogenase *Stenotrophomonas *sp. LM091 WP_070425978	98
pALDR177	768	Xanthine dehydrogenase family protein molybdopterin‐binding sub unit *Rhizobium *sp. Root564 WP_062426820	99
	329	Xanthine dehydrogenase family protein subunit M *Rhizobium *sp. Leaf155 WP_062597871	98
	182	(2Fe−2S)‐binding protein *Rhizobium *sp. WP_062442533	97
pALDSV3	485	Aldehyde dehydrogenase *Pseudomonas *sp. A214 WP_076384861	72
pEGA1	504	Aldehyde dehydrogenase *Microbacterium pygmaeum* WP_091486269	73
pEMMO	484	Benzaldehyde dehydrogenase *Acinetobacter *sp. ANC 3832 WP_086192356	85
pER2AH	491	Aldehyde dehydrogenase *Arthrobacter *sp. Leaf69 WP_056430460	94
pER2AH2	490	Salicylaldehyde dehydrogenase *Arthrobacter *sp. P2b WP_079598892	98
pRG1	501	Aldehyde dehydrogenase family protein *Bacillus sp*. WP_057215027.1	91
pRG2	490	Aldehyde dehydrogenase *Pseudomonas fulva* WP_013791146	90
URAGR	472	Benzaldehyde dehydrogenase *Agrobacterium *sp. SCN 61–19 ODS51427	85
Vmix	490	Phenylacetaldehyde dehydrogenase *Verrucomicrobia *sp. OHE78850	73

To analyze a substrate specificity of the screened enzymes, the bioconversion of substrates by whole cells was monitored by UV‐Vis spectrophotometer and products of the reaction were confirmed by HPLC‐MS analysis (Table 3). For some substrates, the colorimetric assay based on the formation of formazan by the cell‐free extracts was applied (Table 4). Thirteen derivatives of indole‐3‐carboxyaldehyde were tested. The most preferred substrates among the tested ones were 5‐bromindole‐3‐carboxaldehyde, 6‐benzyloxyindole‐3‐carboxaldehyde, and 1*H*‐benzo[g]indole‐3‐carboxaldehyde, which were oxidized by 18 ALDHs (Table 3). Only one strain (pALDR177) could oxidize 2‐phenylindole‐3‐carboxaldehyde. The whole cells with an empty vector (*E. coli* DH5α/pUC19) did not show any activity on the tested substrates, confirming that the ALDHs were encoded by the metagenomic inserts. Even though among aldehydes without indole ring, the favorable substrate was 3‐hydroxybenzaldehyde, which was oxidized by 19 clones, the hits showed very different substrate specificity (Table 4), and hence, the offered screening platform allowed the identification of ALDHs both of different structures and catalytic properties.

**Table 3:** Activity of aldehyde dehydrogenases toward derivatives of indole‐3‐carboxaldehyde

	5BR3C	6B3C	1HB3C	5M3C	T3C	6I3C	BT3C	1M3C	4N3C	5B3C	1,2MHC	4B3C	2P3C
pALDR177	**+**	**+**	**+**	**+**	**+**	**+**	**+**	**+**	**+**	**+**	**+**	**+**	**+**
pALDBS21	**+**	**+**	**+**	**+**	**+**	**+**	**+**	**+**	**+**	**+**	**+**	**−**	**−**
JU61	**+**	**+**	**+**	**+**	**+**	**+**	**+**	**+**	**+**	**+**	**−**	**−**	**−**
pALD442	**+**	**+**	**+**	**+**	**+**	**+**	**+**	**+**	**+**	**+**	**−**	**−**	**−**
pALDGA1	**+**	**+**	**+**	**+**	**+**	**+**	**+**	**+**	**+**	**+**	**−**	**−**	**−**
pALDBSal	**+**	**+**	**+**	**+**	**+**	**+**	**+**	**+**	**+**	**+**	**−**	**−**	**−**
pRG1	**+**	**+**	**+**	**+**	**+**	**+**	**+**	**+**	**+**	**−**	**−**	**−**	**−**
PER2AH2	**−**	**+**	**+**	**+**	**+**	**+**	**+**	**+**	**+**	**+**	**−**	**−**	**−**
pALDMO11	**+**	**+**	**+**	**−**	**+**	**+**	**+**	**+**	**+**	**−**	**−**	**−**	**−**
pEGA1	**−**	**+**	**+**	**+**	**−**	**−**	**+**	**+**	**−**	**+**	**−**	**+**	**−**
pALDMO9	**+**	**+**	**+**	**+**	**+**	**+**	**−**	**−**	**+**	**−**	**−**	**−**	**−**
pER2AH	**+**	**+**	**−**	**−**	**+**	**+**	**+**	**+**	**+**	**−**	**−**	**−**	**−**
Vmix	**+**	**+**	**+**	**+**	**+**	**−**	**−**	**+**	**−**	**−**	**−**	**−**	**−**
pRG2	**+**	**+**	**+**	**+**	**−**	**+**	**−**	**−**	**−**	**−**	**−**	**−**	**−**
pALDSV3	**+**	**−**	**+**	**+**	**+**	**+**	**−**	**−**	**−**	**−**	**−**	**−**	**−**
DON4	**+**	**+**	**+**	**+**	**+**	**−**	**−**	**−**	**−**	**−**	**−**	**−**	**−**
pALDJU6	**+**	**+**	**+**	**+**	**+**	**−**	**−**	**−**	**−**	**−**	**−**	**−**	**−**
pALD458	**+**	**+**	**+**	**+**	**−**	**−**	**−**	**−**	**−**	**−**	**−**	**−**	**−**
pEMMO	**+**	**+**	**−**	**−**	**−**	**+**	**+**	**−**	**−**	**−**	**−**	**−**	**−**
URAGR	**+**	**−**	**+**	**−**	**−**	**−**	**−**	**−**	**−**	**−**	**−**	**−**	**−**

Note

1,2MHC: 1,2‐dimethyl‐indole‐3‐carboxaldehyde; 1HB3C: benzo[g]indole‐3‐carboxaldehyde; 1M3C: 1‐methylindole‐3‐carboxaldehyde; 2P3C: 2‐phenylindole‐3‐carboxaldehyde; 4B3C: 4‐benzyloxyindole‐3‐carboxaldehyde; 4N3C: 4‐nitroindole‐3‐carboxaldehyde; 5B3C: 5‐benzyloxyindole‐3‐carboxaldehyde; 5BR3C: 5‐bromoindole‐3‐carboxaldehyde; 5M3C: 5‐methylindole‐3‐carboxaldehyde; 6B3C: 6‐benzyloxyindole‐3‐carboxaldehyde; 6I3C: 6‐isopropylindole‐3‐carboxaldehyde; BT3C: benzo[b]thiophene‐3‐carboxaldehyde; T3C: 1,6,7,8‐tetrahydrocyclopenta(g)indole‐3‐carboxaldehyde; “+”: the reaction product was observed by the HPLC‐MS analysis and/or by the UV‐VIS spectrum; “−”: no conversion.

**Table 4:** Substrate specificity of aldehyde dehydrogenases

	3HBA	VAN	MFU	FU	DHBA	BA	SA	NA	TCA	PYCA	DMBA	3CHCA	CHCA	FAA	2PHPA
pALDBSal	**+**	**+**	**+**	**+**	**+**	**+**	**+**	**+**	**+**	**+**	**+**	**+**	**+**	**+**	**+**
PER2AH	**+**	**+**	**+**	**+**	**+**	**+**	**+**	**+**	**+**	**+**	**+**	**+**	**+**	**+**	**+**
pALDR177	**+**	**+**	**+**	**+**	**+**	**+**	**+**	**+**	**+**	**+**	**+**	**−**	**+**	**+**	**+**
pALDGA1	**+**	**+**	**+**	**+**	**+**	**+**	**+**	**+**	**+**	**+**	**+**	**+**	**+**	**+**	**−**
pALD442	**+**	**+**	**+**	**+**	**+**	**+**	**+**	**+**	**+**	**+**	**+**	**+**	**+**	**+**	**−**
URAGR	**+**	**+**	**+**	**+**	**+**	**+**	**+**	**+**	**+**	**+**	**+**	**+**	**+**	**+**	**−**
PER2AH2	**+**	**+**	**+**	**+**	**+**	**+**	**+**	**+**	**+**	**+**	**+**	**+**	**+**	**−**	**−**
pRG2	**+**	**+**	**+**	**+**	**+**	**+**	**+**	**−**	**+**	**+**	**+**	**+**	**+**	**+**	**−**
JU61	**+**	**+**	**+**	**+**	**−**	**+**	**+**	**+**	**+**	**+**	**+**	**+**	**+**	**+**	**−**
pEGA1	**+**	**+**	**+**	**+**	**+**	**+**	**+**	**−**	**+**	**+**	**+**	**+**	**+**	**−**	**−**
pALDMO11	**+**	**+**	**+**	**+**	**+**	**+**	**+**	**−**	**+**	**+**	**+**	**+**	**−**	**+**	**−**
pEMMO	**+**	**−**	**+**	**+**	**+**	**+**	**+**	**−**	**+**	**+**	**+**	**+**	**+**	**−**	**−**
pALDSV3	**+**	**+**	**+**	**+**	**−**	**+**	**+**	**−**	**+**	**+**	**−**	**+**	**+**	**+**	**−**
pRG1	**+**	**+**	**+**	**+**	**+**	**+**	**+**	**+**	**−**	**+**	**+**	**−**	**−**	**−**	**−**
DON4	**+**	**−**	**+**	**+**	**+**	**+**	**+**	**+**	**+**	**−**	**−**	**+**	**−**	**+**	**−**
Vmix	**+**	**+**	**+**	**+**	**+**	**+**	**+**	**+**	**−**	**−**	**−**	**−**	**−**	**−**	**−**
pALDMO9	**+**	**+**	**−**	**−**	**+**	**−**	**−**	**+**	**−**	**−**	**+**	**−**	**−**	**−**	**−**
pALD458	**+**	**+**	**+**	**−**	**+**	**−**	**−**	**+**	**−**	**−**	**−**	**−**	**−**	**−**	**−**
pALDBS21	**+**	**+**	**−**	**−**	**+**	**−**	**−**	**+**	**−**	**−**	**−**	**−**	**−**	**−**	**−**
pALDJU6	**−**	**+**	**+**	**+**	**−**	**+**	**−**	**−**	**−**	**−**	**−**	**−**	**−**	**−**	**−**

Note

2PHPA: 2‐phenylpropionaldehyde; 3CHCA: 3‐cyclohexene‐1‐carboxaldehyde; 3HBA: 3‐hydroxybenzaldehyde; BA: benzaldehyde; CHCA: cyclohexanecarboxaldehyde; DHBA: 3,4‐dihydroxybenzaldehyde; DMBA: 4‐(dimethylamino)benzaldehyde; FAA: phenylacetaldehyde; FU: furfural; MFU: 5‐methylfurfural; NA: 1‐naphthaldehyde; PYCA: pyrrole‐2‐carboxaldehyde; SA: salicylaldehyde; TCA: *trans*‐cinnamaldehyde; VAN: vanillin; “+”: The reaction product was observed by the colorimetric assay in the cell‐free extracts of recombinant *Escherichia coli*; “−”: the concentration of the resulting formazan dye was not different from the control.

**Figure 4:**
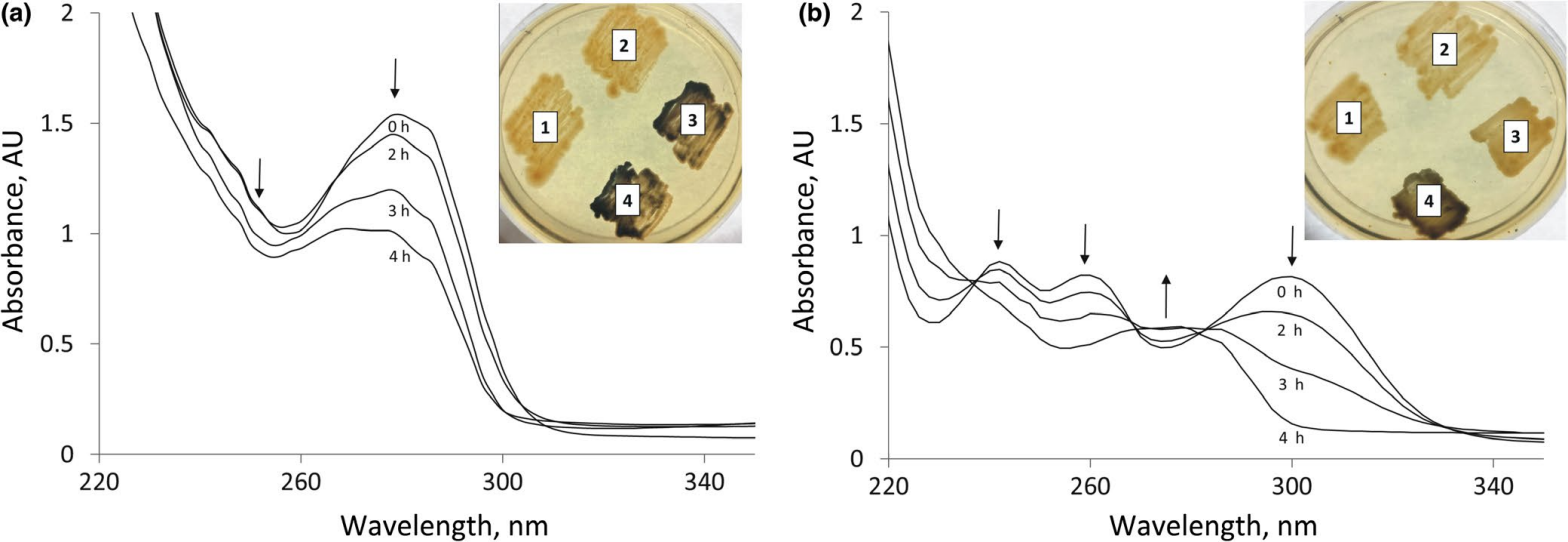
Icm and aldehyde dehydrogenase Vmix activity in the *Rhodococcus erythropolis *SQ1 cells. (a) Consumption of I3CA by cells carrying pNitQC1‐Icm plasmid, (b) bioconversion of I3CA by cells carrying pNitQC1‐KVIA and pNitRT1‐Vmix plasmids. *R. erythropolis *SQ1 cells with different plasmid combinations were plated on I3CA (a) and indole‐3‐carboxaldehyde (b). 1—pNitQC1 + pNitRT1 (two empty vectors), 2—pNitQC1 + pNitRT1‐Vmix (one empty vector and the other one carrying *aldh* gene), 3—pNitQC1‐Icm + pNitRT1 (one empty vector and the other one carrying *icm* gene), 4—pNitQC1‐Icm + pNitRT1‐Vmix (*icm* and *aldh* genes)

To test whether the screening of ALDHs could be carried out in another bacterial host, one ALDH gene was subcloned to the pNitRT1 plasmid for expression in *R. erythropolis* SQ1. It turned out, that the cells transformed with pNitRT1‐Vmix and pNitQC1‐KVIA could produce indigo dye on the plates supplemented with indole‐3‐carboxaldehyde (Figure 4). Considering the fact that not all enzymes encoded in the metagenome can be active in *E. coli* cells, the Gram‐positive host such as *Rhodococcus* sp. would be a good additional alternative for a functional screening of ALDHs, thereby expanding the variety of the selectable enzymes.

**Figure A2:**
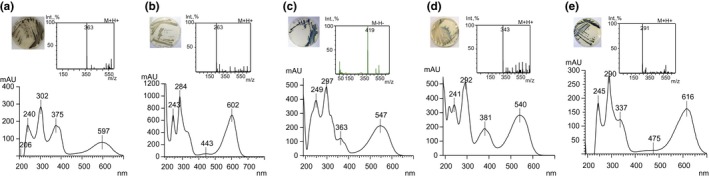
Formation of blue pigment in the presence of Icm and different indole carboxaldehydes. *Escherichia coli* DH5α (pACYC‐KVIA/pALDR177) cells on the plates, UV and Mass spectra of formed compounds: (a) 1*H*‐benzo[*g*]indole‐3‐carboxaldehyde, (b) I3C, (c) 5‐bromindole‐3‐carboxaldehyde, (d) 1,6,7,8‐tetrahydrocyclopenta[*g*]indole‐3‐carboxaldehyde, (e) 5‐methylindole‐3‐carboxaldehyde

To test further the substrate specificity of Icm and to enlarge the list of compounds applicable for the screening purposes, we have chosen *E. coli* cells transformed with pACYC‐KVIA and pALDR177 plasmids. According to the activity tests, the ALDR177 clone was able to oxidize the widest spectrum of derivatives of indole‐3‐carboxaldehydes to the corresponding carboxylic acids. Transformants were spread on the agar plates supplemented with various indole ring containing aldehydes and incubated at 30°C for 48 hr. Colonies remained uncolored on 4‐nitroindole‐3‐carboxaldehyde, 4‐benzyloxyindole‐3‐carboxaldehyde, 5‐benzyloxyindole‐3‐carboxaldehyde, 6‐benzyloxyindole‐3‐carboxaldehyde, benzo[b]thiophene‐3‐carboxaldehyde, however, pigmented colonies appeared on media supplemented with 5‐methylindole‐3‐carboxaldehyde, benzo[g]indole‐3‐carboxaldehyde, 1,6,7,8‐tetrahydrocyclopenta[g]indole‐3‐carboxaldehyde, 5‐bromoindole‐3‐carboxaldehyde (Figure A2), indicating that the corresponding carboxylic acids served as substrates for Icm. The consumption of aldehydes was confirmed by HPLC‐MS. It could be concluded that those aldehydes might be applicable for a more selective screening of ALDHs.

### 3.4 Screening of amidohydrolases

Encouraged with the successful screening of ALDHs, we tested whether *icm* gene‐based approach could be extended for the functional screening of other enzymes. *N*‐(3‐hydroxypropyl)‐indole‐3‐carboxamide was synthesized and used as a substrate for amidohydrolases. One positive clone forming a blue colony was identified after testing two metagenomic DNA libraries (approx. 20,000 clones). The plasmid pMO13 isolated from this hit contained a DNA fragment encoding a 489 aa long protein, which was 90% identical to hypothetical amidase (WP_010677135) and shared 41% identity to indoleacetamide hydrolase (WP_011083078). Subsequently, MO13 amidase was cloned into pET‐21a(+) vector, heterologously expressed in *E. coli* BL21(DE3), and purified as the C‐His_6_‐tagged recombinant protein. The analysis of the substrate specificity of MO13 amidohydrolase showed that in addition to *N*‐(3‐hydroxypropyl)‐indole‐3‐carboxamide, the enzyme could hydrolyze indole‐5‐carboxamide, nicotinamide, hippuric acid, glycyl‐L‐leucine, and L‐valyl‐L‐valine to corresponding carboxylic acids. MO13 was also active toward L‐leucin‐*p*‐nitroanilide, 4‐nitroacetanilide, and 4‐nitrobenzanilide. Moreover, this amidase was able to regioselectively deprotect lysine in *N*_ε_ position when *N_α_*,*N*_ε_‐di‐Z‐L‐lysine or *N_α_*‐Boc‐*N*_ε_‐Z‐L‐lysine was used as substrates.

## 4 CONCLUSIONS

In this study, we have successfully identified a monooxygenase (Icm) active toward indole‐3‐carboxylic acid. The indigo formation due to activity of Icm allowed the development of a simple system for functional screening of enzymes from the metagenomic libraries. We showed that different enzymes, for example, ALDHs or amidohydrolases could be identified depending on the used substrate. Moreover, the system might be easily extended for screening other activities as shown in Figure 2. The only requirement is that the product of enzymatic reaction would be indole‐3‐carboxylic acid (with or without substituents in the indole ring), which could be a substrate for an auxiliary enzyme Icm. It should be noted that Icm was active not only in *E. coli* but also in *R. erythropolis* SQ1 cells that could open additional possibilities to use the different bacterial hosts for the functional screening.

## CONFLICT OF INTEREST

The authors declare no conflict of interests.

## AUTHORS CONTRIBUTION

VČ, MS, JV, and RoM designed the experiments. VČ, MS, JV, RG, RiM, IS, MS, JJ, and DT performed the experiments. All authors analyzed the data. VČ, MS, and JV wrote the manuscript. All authors read the final manuscript.

## ETHICS STATEMENT

None required.

## Data Availability

All DNA sequences are submitted to GenBank. The sequences obtained in the present study were deposited to the GenBank database under the accession numbers MG770119–MG770138, MG786188, MG786189, MG775032, MK284926 and MH476458. The full list is given in Table A2. All data generated or analyzed during this study are included in this published article.

## APPENDIX 1

**Table A1:** Source of chemicals

Aldrich, Buchs, Switzerland
5‐Methylfurfural, 1‐naphthaldehyde, 3‐cyclohexene‐1‐carboxaldehyde, 3‐hydroxybenzaldehyde, *trans*‐cinnamaldehyde, vanillin, phenylacetaldehyde, furfural, 3,4‐dihydroxybenzaldehyde, benzaldehyde, salicylaldehyde, pyrrole‐2‐carboxaldehyde, 4‐(dimethylamino)benzaldehyde, cyclohexanecarboxaldehyde, 1,2‐dimethyl‐1H‐I3C, 5‐methylindole‐3‐carboxaldehyde, 4‐nitroindole‐3‐carboxaldehyde, 1*H*‐benzo[*g*]indole‐3‐carboxaldehyde, 2‐phenylindole‐3‐carboxaldehyde, 6‐izopropylindole‐3‐carboxaldehyde, nicotinic acid, 2‐picoline acid, 4‐picoline acid, indole‐6‐carboxylic acid, 5‐nitroindole‐3‐carboxylic acid, salicylic acid, 2‐phenylpropionaldehyde, 1,6,7,8‐tetrahydrocyclopenta(g)indole‐3‐carboxaldehyde, 5‐bromindole‐3‐carboxaldehyde, 5‐benzyloxyindole‐3‐carboxaldehyde, 1‐methylindole‐3‐carboxaldehyde, 4‐nitroacetanilide, 4‐nitrobenzanilide, 1*H*‐indole‐5‐carboxamide, hippuric acid, *N,N,N′,N*′‐tetramethyl‐*O*‐(1*H*‐benzotriazol‐1‐yl)uronium hexafluorophosphate (HBTU), dimethylformamide NADH, FAD
Combi Blocks, SanDiego, USA
4‐Benzyloxyindole‐3‐carboxaldehyde, 6‐benzyloxyindole‐3‐carboxaldehyde, benzo[b]thiophene‐3‐carboxaldehyde, 5‐hydroxy pyrazine‐2‐carboxylic acid, indoline‐2‐carboxylic acid, indole‐4‐carboxylic acid, indole‐5‐carboxylic acid, indole‐7‐carboxylic acid
Fluka, Steinheim, Switzerland
Indole‐2‐carboxylic acid, nicotinamide, L‐leucin‐*p*‐nitroanilide.
Sigma, St. Louis, USA
7‐Methylindole‐3‐carboxylic acid, indole 3‐carboxylic acid (I3CA), indole‐3‐carboxaldehyde (I3C)
Reanal, Budapest, Hungary
Glycyl‐L‐leucine, L‐valyl‐L‐valine
Merck, Darmstadt, Germany
3‐Amino‐1‐propanol, z‐lys(z)‐OH, boc‐lys(z)‐OH, ethyl acetate, triethylamine
Thermo Fisher Scientific Vilnius, Lithuania
5‐Bromo‐4‐chloro‐indolyl‐β‐D‐galactopyranoside (X‐gal), isopropyl‐β‐D‐thiogalactopyranoside (IPTG)

**Table A2:** Clones from soil metagenomic libraries

Clone name	GenBank accession number
JU61	MG770119
pER2AH2	MG770120
pALDR177	MG770121
pER2AH	MG770122
VMIX	MG770123
pALDSV3	MG770124
pALDMO9	MG770126
pALDBSal	MG770127
pALDBS21	MG770128
pALDMO11	MG770129
pEGA1	MG770130
pEMMO	MG770131
pALD458	MG770132
URAGR	MG770133
pRG1	MK284926
pRG2	MG770134
pALDJU6	MG770135
DON4	MG770136
pALDGA1	MG770137
pALD442	MG770138
pMILC	MG786188
pNVS	MG786189
pKVIABam8	MG770125
KVIA (16S RNA gene)	MG775032
MO13	MH476458

**Table A3:** Solubility of Icm at different induction conditions in *Escherichia coli*. Condition A—induction for 4 hr with 0.5 mM IPTG at 30°C, condition B—induction for 4 hr with 0.05 mM IPTG at 30°C, and condition C—overnight induction with 0.05 mM IPTG at 16°C. For Strep‐Tag MBP‐His_6_‐KVIA, anhydrotetracycline (200 μg/L) was used instead of IPTG. ND: not detected

	Condition A			Condition B			Condition C		
	Total amount of Icm (mg/L)	Amount of soluble Icm (mg/L)	% soluble	Total amount of Icm (mg/L)	Amount of soluble Icm (mg/L)	% soluble	Total amount of Icm (mg/L)	Amount of soluble Icm (mg/L)	% soluble
Icm	10.25	ND	ND	5.9	ND	ND	11.7	ND	ND
His_6_‐Icm	14.9	ND	ND	11.1	ND	ND	19.1	ND	ND
His_6_‐MBP‐His_6_‐Icm	19.6	0.4	1.9	18.3	6.8	37.3	44.4	9	20.2
Strep‐Tag MBP‐His_6_‐Icm	39.2	3.3	8.4	29.1	7.3	24.9	35	21.4	61
